# The Role of Lymph Node Dissection in the Management of Upper Urothelial Cancer: A Nodal Status-Based Meta-Analytical Study

**DOI:** 10.5152/tud.2023.23045

**Published:** 2023-11-01

**Authors:** Abdalla Ali Deb, Ayman Agag, Naufal Naushad, Alice Hartley, Hosam Serag

**Affiliations:** 1 Department of Urology, James Cook University Hospital, Middlesbrough, UK; 2 Department of Urology, Frimley Park Hospital, Camberley, UK; 3 Department of Urology, North Tees University Hospital, Stockton, UK; 4 Department of Urology, South Tyneside and Sunderland NHS Foundation Trust, Sunderland, UK; 5 Department of Urology, University Hospitals Birmingham, Birmingham, UK

**Keywords:** UTUC, upper tract urothelial cancer, nephroureterectomy, lymph node dissection, lymphadenectomy

## Abstract

This systematic review was performed to study the prognostic value of lymph node dissection (LND) during nephroureterectomy in upper tract urothelial cancer (UTUC). Five databases were searched on September 11, 2022, to include studies that compared whether LND was performed, the extent of dissection (complete vs. incomplete), and the nodal status (positive “pN+” vs. negative “pN0”). Outcomes included prognosis (overall survival “OS,” cancer-specific survival “CSS,” disease-free survival “DFS,” and recurrence-free survival “RFS”) and complications. High-grade complications (≥ grade 3 according to the Clavien–Dindo classification). Data analysis were conducted through STATA. The pooled data are reported log odds ratio (logOR) with 95% CI. Thirty-three studies were analyzed. The LND resulted in improved 5-year OS [logOR = 0.10; 95% CI: 0.06-0.15], 5-year CSS [logOR = 0.10; 95% CI: 0.04- 0.17], and 10-year CSS [logOR = 0.14; 95% CI: 0.06-0.21] when compared to non-LND. However, LND was associated with greater risk of high-grade complications [logOR = 0.62; 95% CI: 0.26-0.98]. Complete LND was associated with lower risk of cancer-specific mortality than incomplete LND [logOR = −0.69; 95% CI: −1.22-−0.16]. The pN0 patients had better 5-year OS; however, pN+ patients had better prognosis in DFS, RFS (at 2 and 5 years), and CSS (at 2, 5, and 10 years). Lymph node dissection provides a protective role in terms of 5-year OS and 5-year and 10-year CSS among UTUC patients. However, it is associated with higher risk of high-grade complications. The extent of dissection plays a minor prognostic role, while the positivity of resected nodes has great prognostic value in UTUC.

**Main Points**

- Upper tract urothelial cancer (UTUC), although rare, is associated with poorer prognosis when compared to bladder cancer.
- Nephroureterectomy is the standardized treatment for UTUC patients. The prognostic role of lymph node dissection during nephroureterectomy is not confirmed, particularly due to the lack of randomized trials.
- Lymph node dissection is associated with better prognosis regarding overall and cancer-specific survival.
- The extent of dissection (complete vs. incomplete) has a prognostic role only in cancer-specific survival.
- Node-positive disease is associated with improved survival in terms of disease-free, recurrence-free, and cancer-specific survival.

## Introduction

Unlike bladder cancer, upper tract urothelial cancer (UTUC) is considered a relatively rare carcinoma, accounting for around 5% to 10% of all cancers originating from the urothelial system.^1^ Based on the recent cancer statistics from the United States, there are 4010 new cases of UTUC annually, while bladder cancer accounted for 81 180 new cases in 2022.^2^ Despite having a lower incidence rate, UTUC is frequently diagnosed late, and patients usually present with advanced disease, therefore having a poorer prognosis.^3^

Nephroureterectomy is the standardized management approach for high-risk UTUC regardless of the site of the tumor. This procedure involves the removal of the kidney, the entire ureter, and the bladder cuff. In instances where the bladder cuff is not completely excised, the risk of bladder cancer recurrence becomes considerably higher.^1^ Lymph nodal metastasis is known as an independent risk factor for poorer outcomes.^4,5^ Therefore, it has been suggested that removal of the lymph nodes, could play an additional protective role in UTUC patients undergoing nephroureterectomy to allow better survival outcomes for patients compared to those who did not undergo lymph node dissection (LND)—no lymphadenectomy (pNx).^1,6^ To date the reported findings are contradictory and are mainly based on retrospective cohort/chart-review studies.^7-15^

Recent empirical evidence suggests a correlation between the presence of positive dissected lymph nodes and prognostic outcomes following nephroureterectomy in UTUC patients. Some reports have indicated that UTUC patients with positive lymph nodes (pN+) have poorer survival outcomes when compared to those with negative lymph nodes (pN0),^9,10,16^ while others have shown superiority of LND in pN+ when compared to pN0.^17,18^ Moreover, the extent of LND has also been suggested to play a role in predicting the prognosis of UTUC in patients undergoing nephroureterectomy, although there is no clear consensus.^12-14,19^

Because of the lack of clarity regarding the role of LND in nephroureterectomy among UTUC patients, we aimed to conduct this systematic review and meta-analysis to determine the role of LND on the survival of this patient population by pooling relevant data from all available evidence. In addition, we examined the role of LND under specific circumstances (node-positive vs. node-negative patients and complete vs. incomplete dissection).

## Material and Methods

### Study Design

This review was done as per the Preferred Reporting Items for Systematic Reviews and Meta-Analysis (PRISMA) recommendations for conducting meta-analyses. A protocol in priori was not registered on PROSPERO or other protocol registries as it is not mandated by recent recommendations.^20^ We followed the PICOS criteria in conducting this study. Our population included patients with upper urothelial cancer, our intervention was LND, our comparison was no LND, our outcomes included patients’ prognosis/survival, and the design of included studied ranged from observational to experimental studies.

### Search Strategy

On September 11, 2022, we searched several databases, namely PubMed, Scopus, Web of Science, Cochrane Registry of Clinical Trials, and Google Scholar. We searched for studies comparing the outcomes of LND during nephroureterectomy in patients with upper urothelial cancer to controls (those who did not undergo LND). As per the recent guidelines,^20^ we retrieved the first 200 studies from Google Scholar to avoid including irrelevant articles. We used different keywords and terms to retrieve relevant papers: (lymphadenectomy OR “lymph node excision” OR “lymph node dissection” OR lymphadenectomies) AND (“Upper tract urothelial cancer” OR nephroureterectomy). A list of other used keywords/terms in the literature was pooled and used in the detailed search query used in every database (Supplementary Table 1). In addition, we used Medical Subject Headings terms in PubMed to avoid missing relevant articles. The database search was updated on November 1, 2022, to include any newly published relevant studies.

Furthermore, we carried out a manual search process where we (1) screened the citations of finally included studies (following the screening stage), (2) searched “similar articles” to finally included studies on PubMed, and (3) searched Google Engine for relevant studies using a set of keywords “nephroureterectomy” + “lymph node.” 

### Study Outcomes

Our primary outcome was to compare the prognosis of UTUC patients among those who underwent and did not undergo LND. The prognosis included cancer-specific survival (CSS), overall survival (OS), disease-free survival (DFS), disease-specific survival (DSS), recurrence-free survival (RFS), all-cause mortality, cancer-specific mortality, recurrence, overall/any complications, complications based on the Clavien–Dindo classification system, and reoperation. Secondary outcomes included comparing the prognosis between various sets of patients based on these nodal status as follows: (1) complete vs. incomplete LND and (2) positive (pN+) vs. negative (pN0) lymph nodes (LNs). All outcomes were presented based on the timing of the follow-up period (years).

### Eligibility Criteria

Our inclusion criteria included (a) observational and experimental studies that included patients with (a) upper urothelial cancer, (b) comparing LND to non-LND (either complete or incomplete) in node-positive or node-negative patients, and (c) reporting the prognosis of such patients in terms of survival and complications.

We ruled out studies during screening if they had at least 1 of these criteria: (a) nonoriginal studies (secondary research, letters to editors, comments, guidelines, etc.), (b) studies including patients with other types or locations of cancer, (c) studies lacking a comparison group or not involving patients who underwent LND, (d) studies examining outcomes other than the above-mentioned ones, (e) studies reporting qualitative data, and (f) duplicate research studies or those with overlapping patients’ data.

### Study Selection

Records were retrieved from searched databases and then imported into EndNote Software, where duplicate studies were removed automatically. Then, the remaining studies were exported into an Excel sheet for the actual screening process. The screening of retrieved citations was carried out in 2 steps: (1) title/abstract and (2) full-text screening. After completing the first step, we retrieved the full papers of potentially eligible studies. Articles were then screened against our previously-mentioned criteria. The decision of including or excluding a study was not based on its language, country, or year of investigation. Two of the study authors were involved in this step, and if any differences were found between them, the corresponding author’s opinion was sought. This accounted for 23 articles of those included in this review.

### Data Extraction

The formal data extraction was done following a pilot phase to determine the outcome endpoints and any subgroups involved. Then, an Microsoft Excel sheet was designed. This sheet was made up of 2 domains. The first domain was designed to extract the baseline data of included records (authors’ names, year of investigation, country of investigation, study design, and the duration of follow-up] and patients [sample size, tumor histology and location, type of surgery, LN category, age, and gender]. The second domain was designed for the outcomes data [OS, CSS, DFS, DSS, RFS, recurrence, complication categories based on the Clavien–Dindo classification system, any complications, and reoperation]. Two researchers carried out the data extraction from individual studies. Finally, extracted data were checked for accuracy among review authors through regular group meetings with the senior author.

### Quality Assessment

All of the analyzed studies involved retrospective cohorts; thus, we used the Newcastle Ottawa Scale for observational studies to assess the risk of bias associated with the methodology of these studies. The assessment included 3 main aspects: selection (4 parts), comparability (2 parts), and outcome reporting (3 parts). The overall quality was deemed either good, fair, or poor based on the overall scoring of each study. This step was done by 2 of the review authors and was revised by the corresponding author to ensure accurate results.

### Data Synthesis

We used STATA (Version 16) to run our meta-analyses. The metan command was used to pool the log odds ratio (logOR) and its corresponding 95% CI. The choice of the statistical model was dependent upon the observation of statistical heterogeneity. For instance, in instances where heterogeneity was observed, the random-effects model was used. If statistical heterogeneity was absent, the fixed-effects model was selected. As for method selection, the restricted maximum likelihood method was selected when a continuous outcome was analyzed and significant heterogeneity was observed. In the case of dichotomous data, the Mantel–Haenszel method used. Statistical heterogeneity was deemed significant if the *I^2^* value was >50% or if its *P *value was below the cutoff point of .05. A subgroup analysis was carried out according to the follow-up period (years). When heterogeneity was encountered, we conducted sensitivity analysis where studies were ruled out one at a time to determine whether or not the reported effect size would differ. Noteworthy, we could not assess the risk of publication bias because the number of included studies in each analysis was lower than the minimal required number to run this analysis (<10 studies). 

## Results

### Search Results

**Figure 1.**
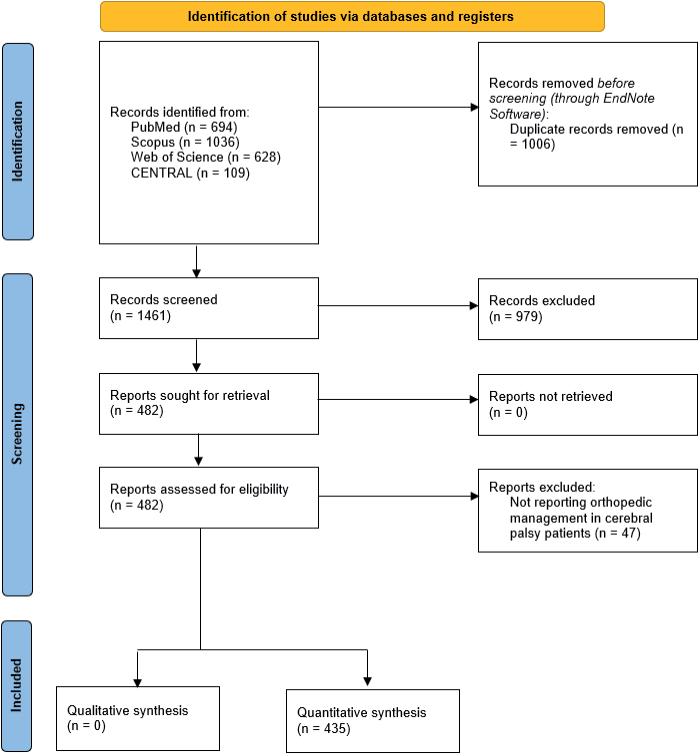
The PRISMA flow diagram of the database search and screening processes.

The results of the initial and updated database search as well as the manual search are presented in Figure 1. Following the completion of database search, 2079 citations were identified. Of those, 565 citations were identified as duplicates through EndNote and, therefore, were excluded before the beginning of the screening process. The first step of screening resulted in 55 potentially relevant articles, and the full texts of these papers were retrieved. Finally, 33 studies were found relevant to our search question and were included for further data synthesis. Meanwhile, 22 studies were ruled out for 2 reasons: the lack of a comparison group (n = 20) and duplicated records (n = 2). The manual and updated database search yielded no additional studies. Overall, 33 studies^7-19,21-40^ were eligible for meta-analysis.

### Baseline Characteristics of Included Studies

The detailed description of the characteristics of analyzed studies in our review is provided in Table 1. Twenty-six studies were retrospective single-center cohorts while 7 studies were retrospective multicenter studies. The number of upper urothelial cancer patients in each study ranged from 60 to 16 619 patients. In total, there were 53 190 patients, of whom 14 178 underwent LND (2323 patients in the pN+ and 8282 patients in the pN0 groups) and 38 833 who did not. The majority of studies were conducted in Japan (n = 17), followed by the United States (n = 4), Canada (n = 2), China (n = 2), Korea (n = 1), Denmark (n = 1), France (n = 1), Germany (n = 1), and Austria (n = 1). The histology and location of urothelial cancer as well as the type of surgery are presented in Table 1. The age and gender of the included upper urothelial cancer patients stratified by the LN status are summarized in Table 2.

### Risk of Bias

The detailed assessment of the quality of analyzed studies is reported in (Table 3). Eleven studies had fair quality (moderate risk of bias), while the remaining 22 studies had poor quality (high risk of bias). None of the included studies were of good quality. Given their retrospective nature, most studies did not control the confounding effect of other covariates either in the design phase (i.e., matching or stratification) or analysis phase (i.e., regression analysis) and did not account for any confounding effect.

## Lymph Node Dissection vs. Non-Lymph Node Dissection

### Prognosis

**Figure 2.**
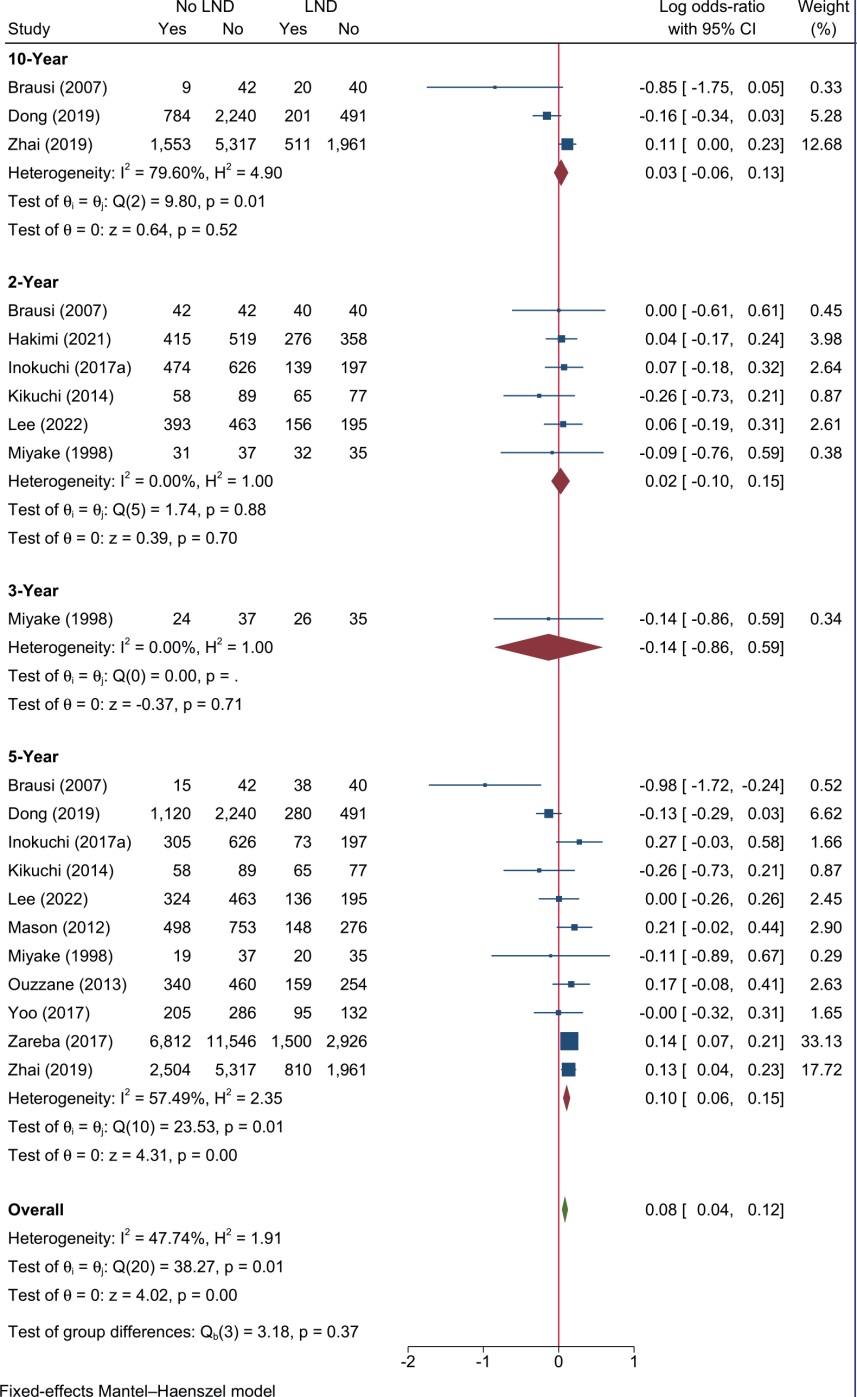
A forest plot showing the odds of overall survival between lymph node dissection and non-lymph node dissection groups stratified by follow-up. LND, lymph node dissection.

Twelve studies reported the OS of patients undergoing LND. The meta-analysis revealed a beneficial impact of LND on the 5-year OS [logOR = 0.10; 95% CI: 0.06-0.15; *I*^*2*^ = 57.49%] when compared to those who did not undergo LND. However, no significant difference in the 2- and 10-year OS was noted between both intervention groups (Figure 2). 

**Supplementary Figure 1.**
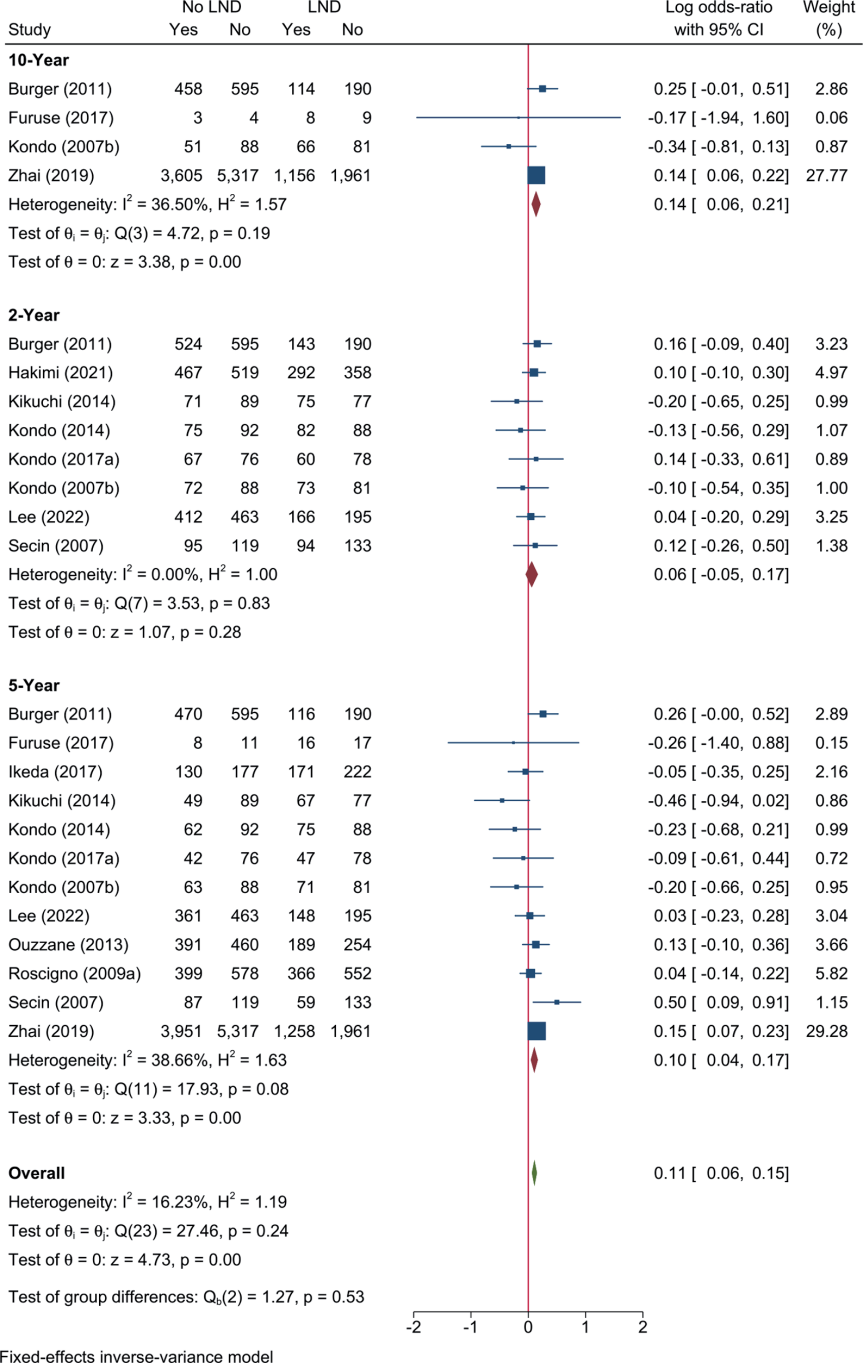
A forest plot showing the odds of cancer-specific survival between LND and non-LND groups stratified by follow-up.

Fourteen studies were included in the analysis of CSS. Although LND did not result in a significant improvement of CSS at 2 years, it showed a beneficial impact on the 5-year [logOR = 0.10; 95% CI: 0.04-0.17; *I*^*2*^ = 38.66%] and 10-year CSS [logOR = 0.14; 95% CI: 0.06-0.21; *I*^*2*^ = 36.50%] when compared to the non-LND group, respectively (Supplementary Figure 1).

**Supplementary Figure 2.**
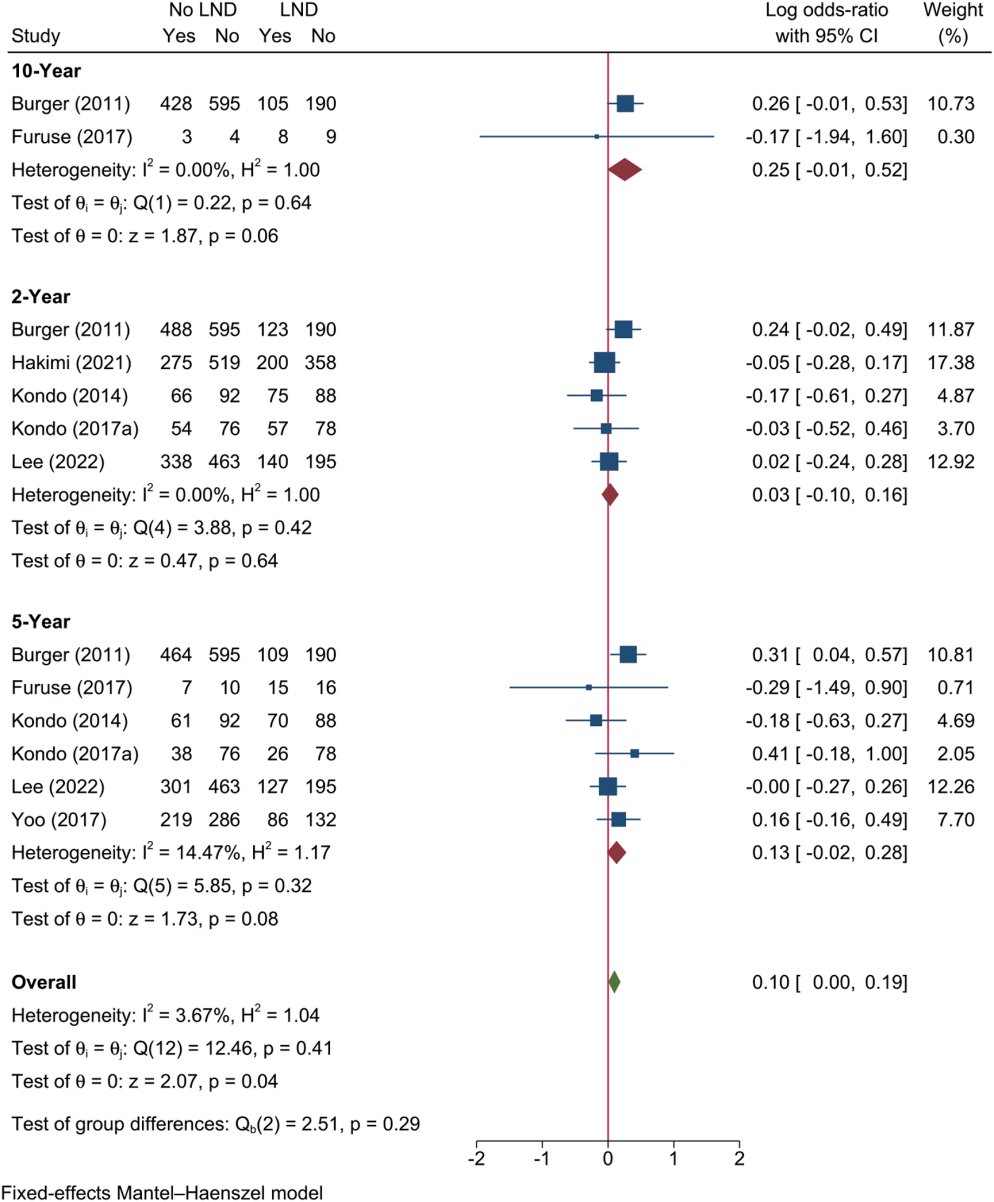
A forest plot showing the odds of recurrence-free survival between LND and non-LND groups stratified by follow-up.

In terms of RFS, our meta-analysis of 7 studies revealed no significant change between LND and non-LND groups at 2 years [logOR = 0.03; 95% CI: −0.10-0.165; *I*^*2*^ = 0%], 5 years [logOR = 0.13; 95% CI: −0.02-0.28; *I*^*2*^ = 14.47%], and 10 years [logOR = 0.25; 95% CI: −0.01-0.52; *I*^*2*^ = 0%] (Supplementary Figure 2).

**Supplementary Figure 3.**
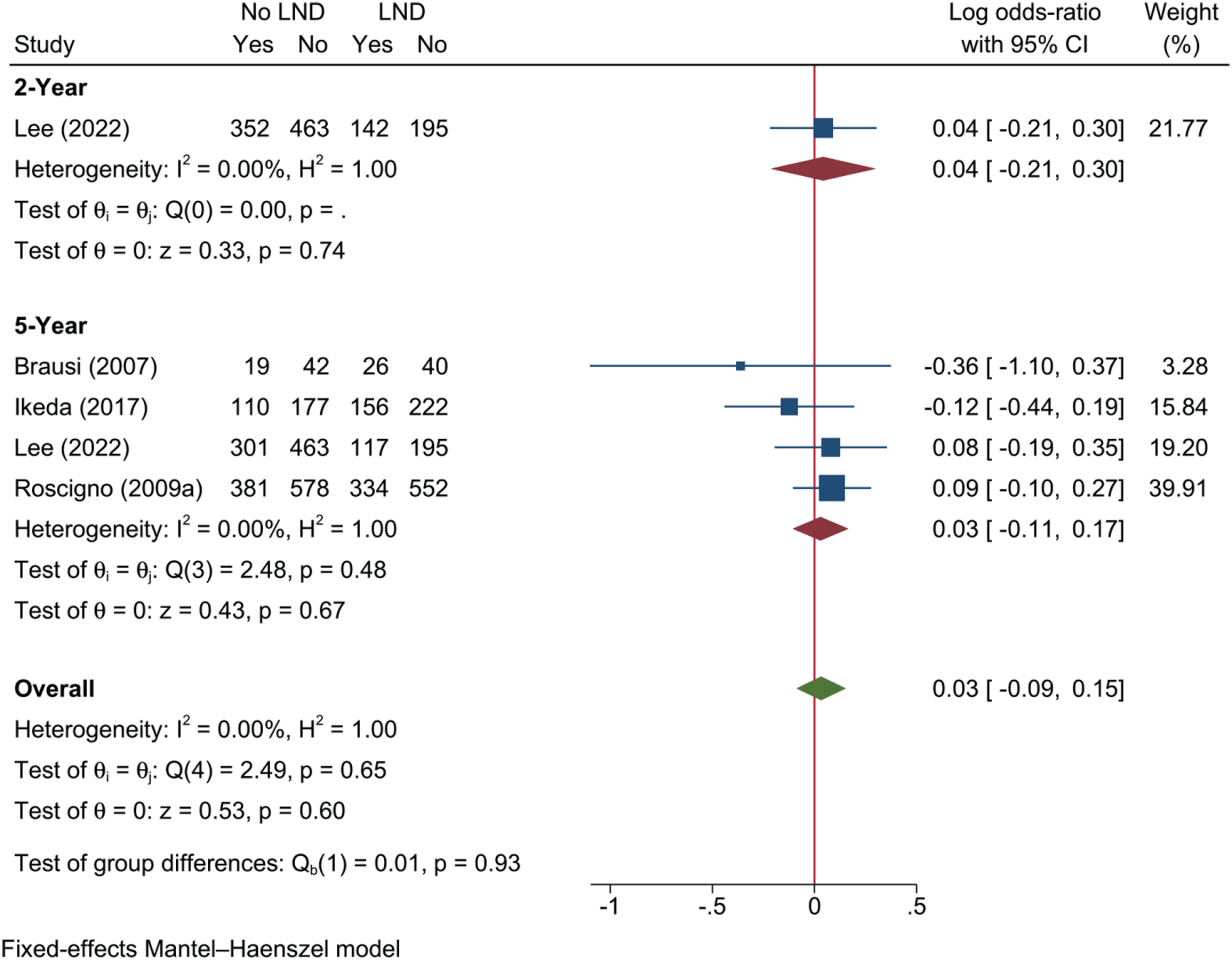
A forest plot showing the odds of disease-free survival between LND and non-LND groups stratified by follow-up.

**Supplementary Figure 4.**
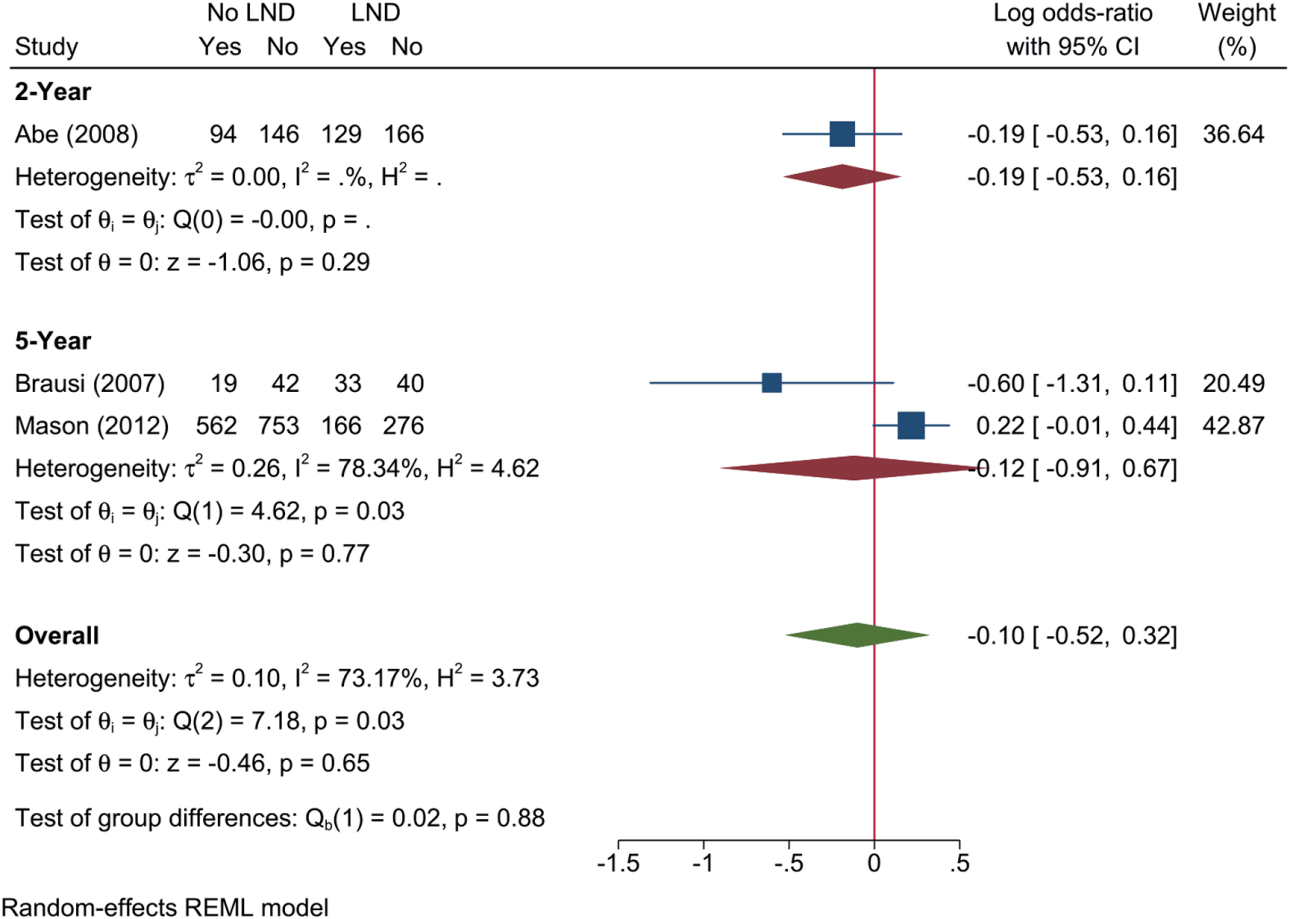
A forest plot showing the odds of any complications between LND and non-LND groups stratified by follow-up.

A similar observation was noted regarding DFS, where the meta-analysis of 4 studies did not highlight any significant different between both groups at 2 and 5 years [logOR = 0.03; 95% CI: −0.11-0.17; *I*^*2*^ = 0%] (Supplementary Figure 3). This is consistent with our observation of DSS at 2 and 5 years where no significant difference in DSS was noted between LND and non-LND groups (Supplementary Figure 4).

### Complications

**Figure 3.**
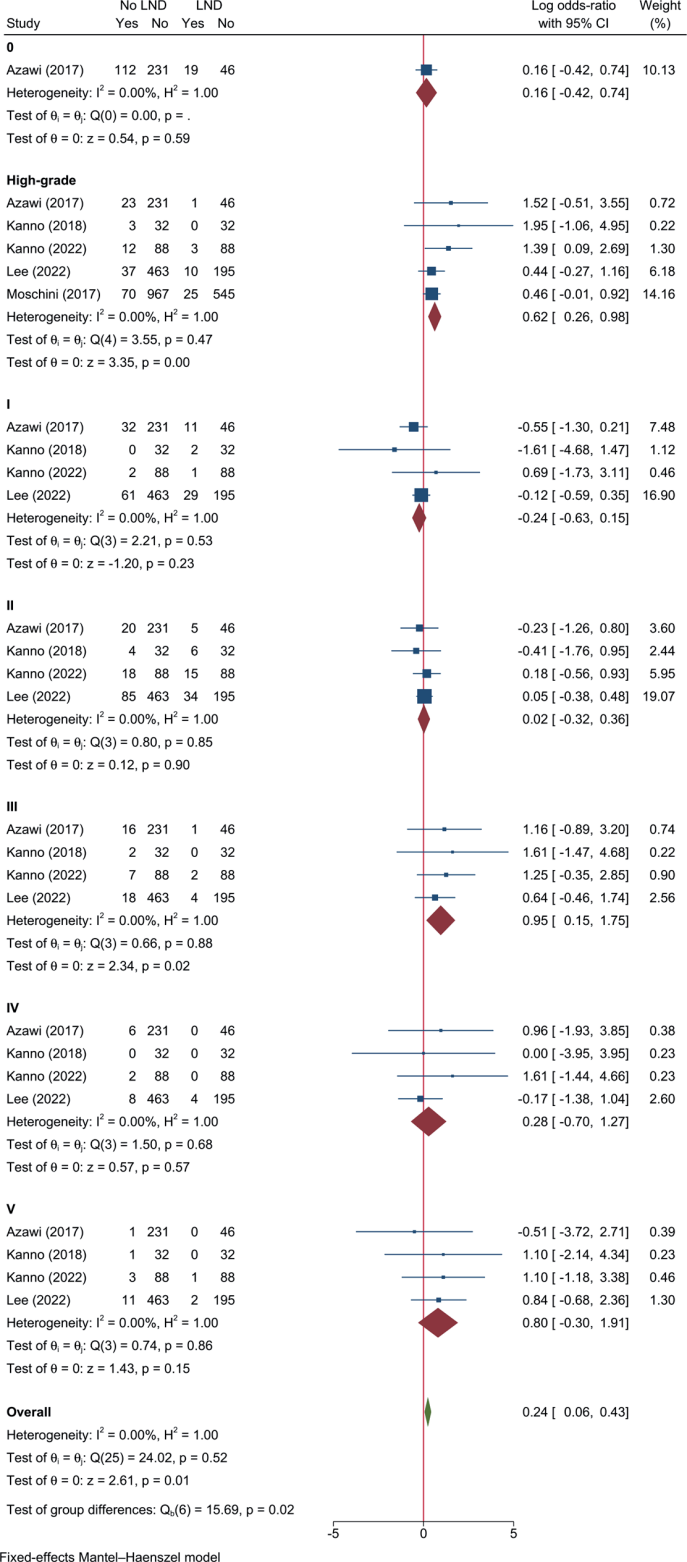
A forest plot showing the odds of complications between lymph node dissection and non-lymph node dissection groups stratified by the Clavien– Dindo classification system. LND, lymph node dissection.

**Supplementary Figure 5.**
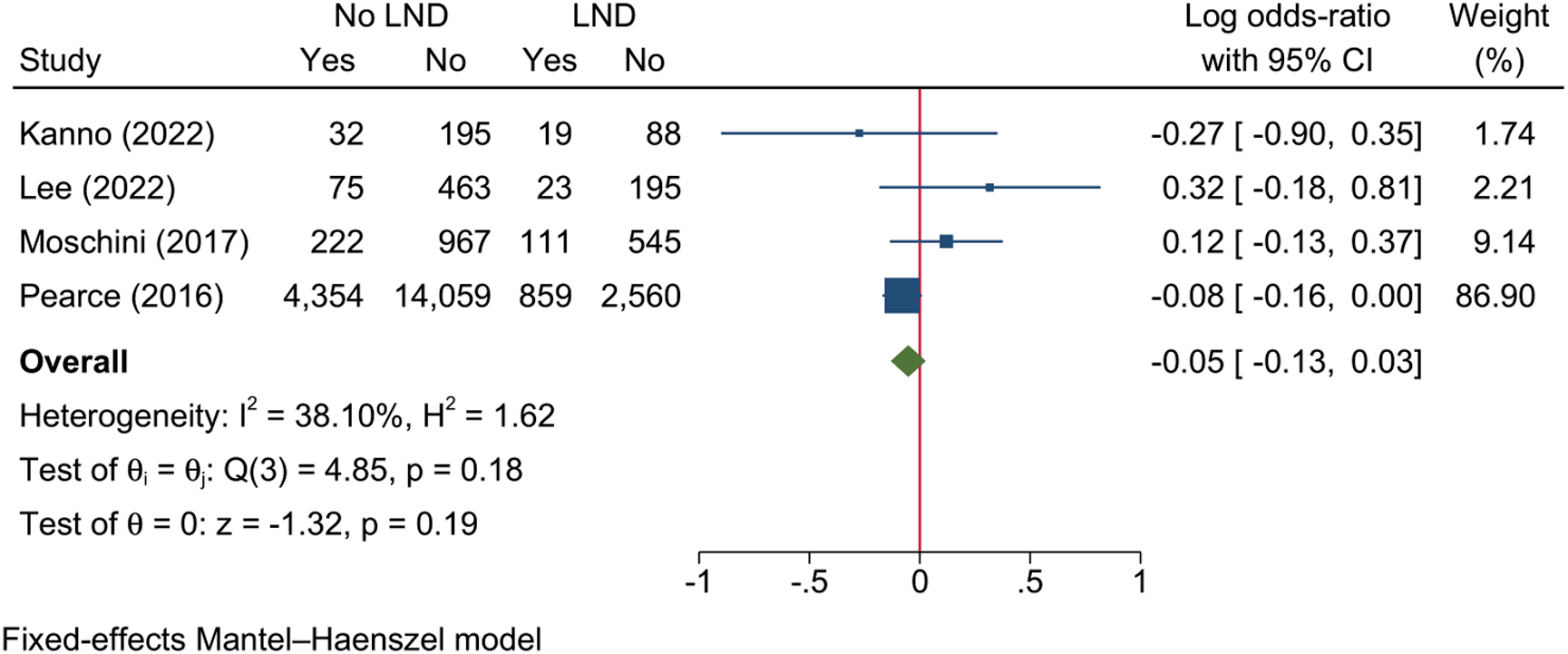
A forest plot showing the odds of overall survival between LND and non-LND groups.

Four studies assessed the complications following LND in upper urothelial cancer patients. Overall, no significant difference was noted between LND and non-LND groups [logOR = −0.05; 95% CI: −0.13-0.03; *I*^*2*^ = 38.10%] (Supplementary Figure 5). However, the analysis of complications based on the Clavien–Dindo classification system revealed a higher risk of high-grade complications (≥ grade III) in the LND group as compared to the non-LND group [logOR = 0.62; 95% CI: 0.26-0.98;* I*^*2*^ = 0%] (Figure 3). 

**Figure 4.**
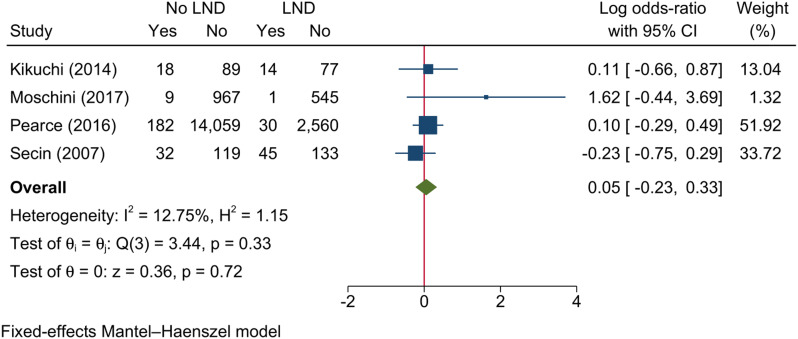
A forest plot showing the odds of all-cause mortality between lymph node dissection and non-lymph node dissection groups. LND, lymph node dissection.

**Figure 5.**
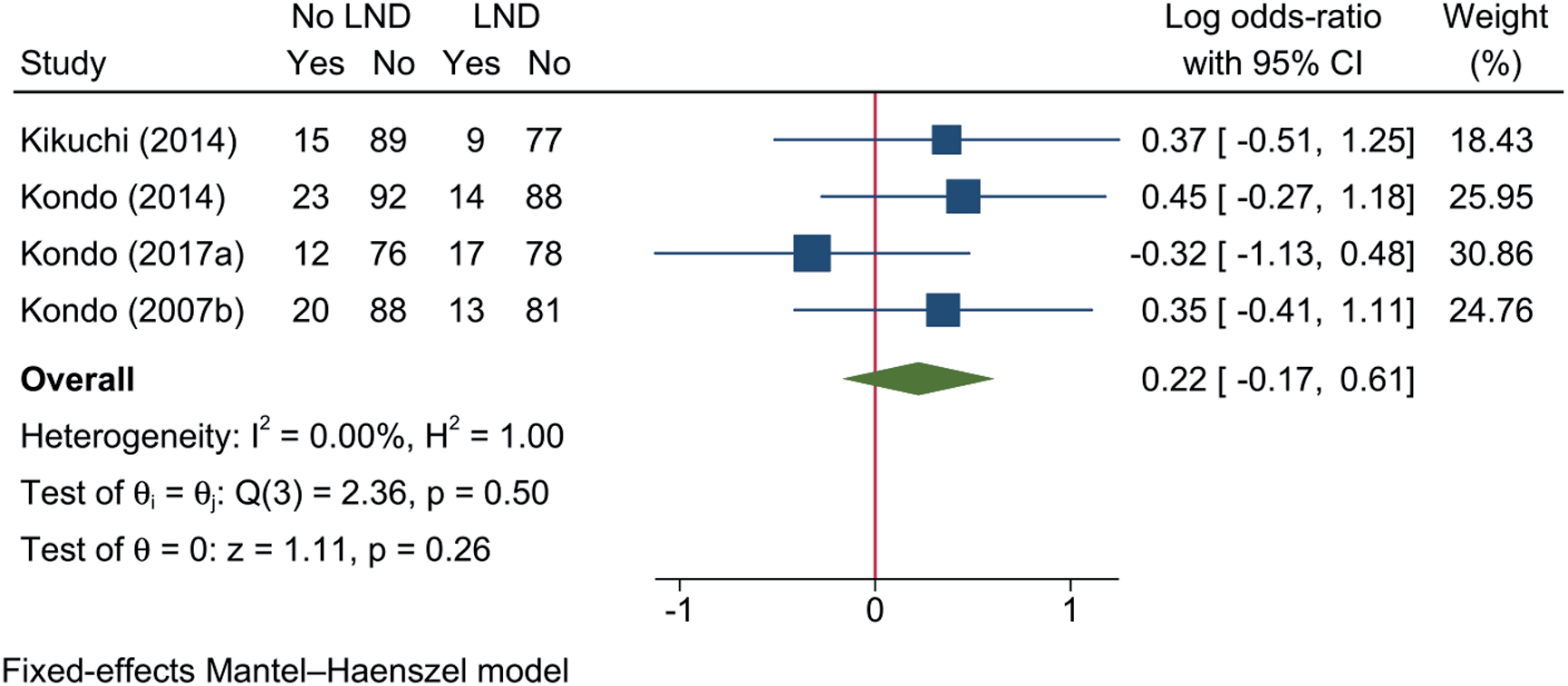
A forest plot showing the odds of cancer-specific mortality between lymph node dissection and non-lymph node dissection groups. LND, lymph node dissection.

All-cause mortality and cancer-specific mortality were reported in 4 studies. The meta-analysis revealed no significant difference in the risk of all-cause mortality [logOR = 0.05; 95% CI: −0.23-0.33; *I*^*2*^ = 12.75%] and cancer-specific mortality [logOR = 0.22; 95% CI: −0.17-0.61; *I*^*2*^ = 0%] between LND and non-LND groups, respectively (Figures 4 and 5).

**Supplementary Figure 6.**
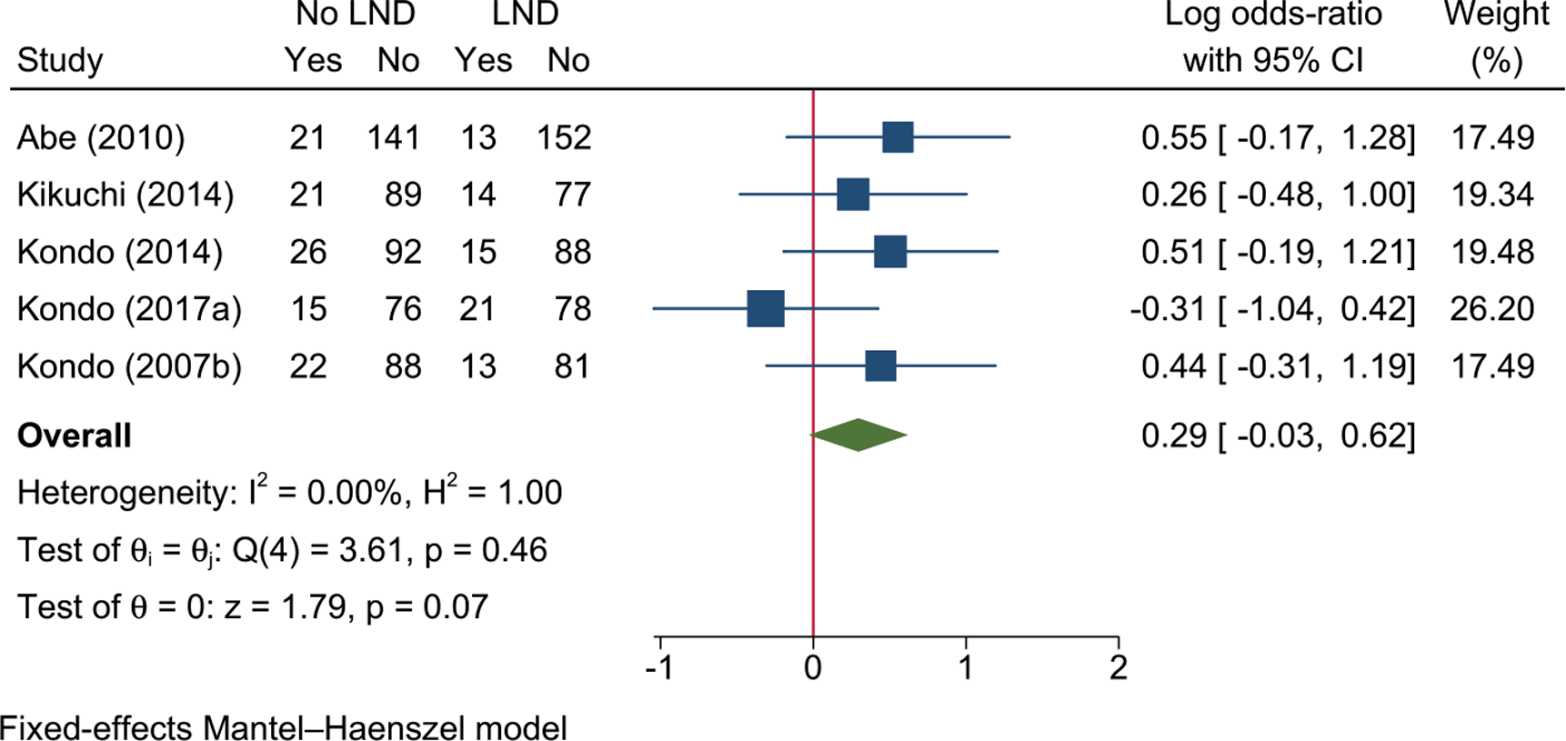
A forest plot showing the odds of recurrence between LND and non-LND groups.

**Supplementary Figure 7.**
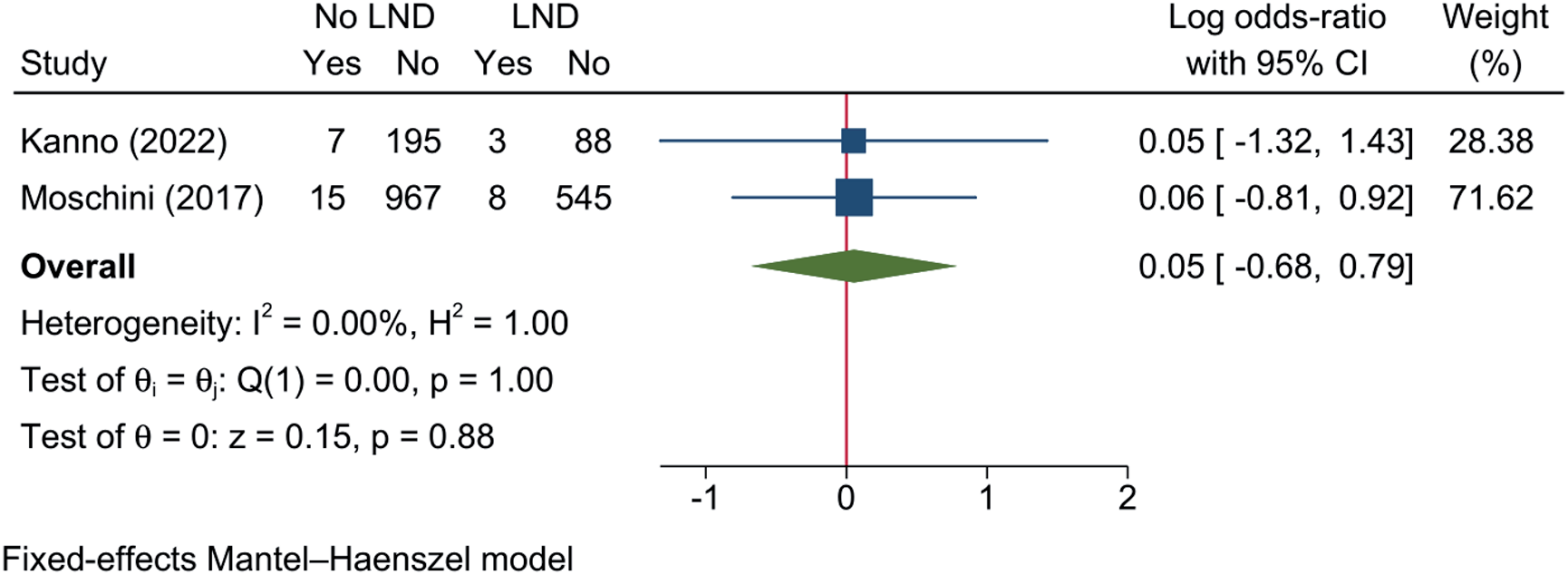
A forest plot showing the odds of reoperation between LND and non-LND groups.

Recurrence was assessed in 5 studies; however, our meta-analysis showed no significant difference between LND and non-LND groups [logOR = 0.29; 95% CI: −0.03-0.62; *I*^*2*^ = 0%] **(** Supplementary Figure 6). Meanwhile, the rate of patients requiring reoperation was reported in only 2 studies. The meta-analysis revealed no significant difference in the risk of reoperation between both intervention groups [logOR = 0.05; 95% CI: −0.68-0.79; *I*^*2*^ = 0%] (Supplementary Figure 7).

## Complete vs. Incomplete Lymph Node Dissection 

### Prognosis

**Supplementary Figure 8.**
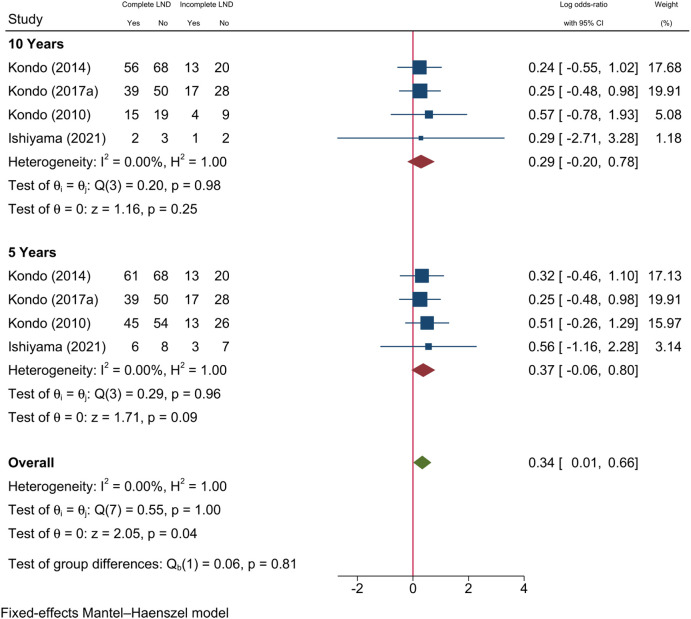
A forest plot showing the odds of cancer-specific survival between complete and incomplete LND stratified by follow-up.

**Supplementary Figure 9.**
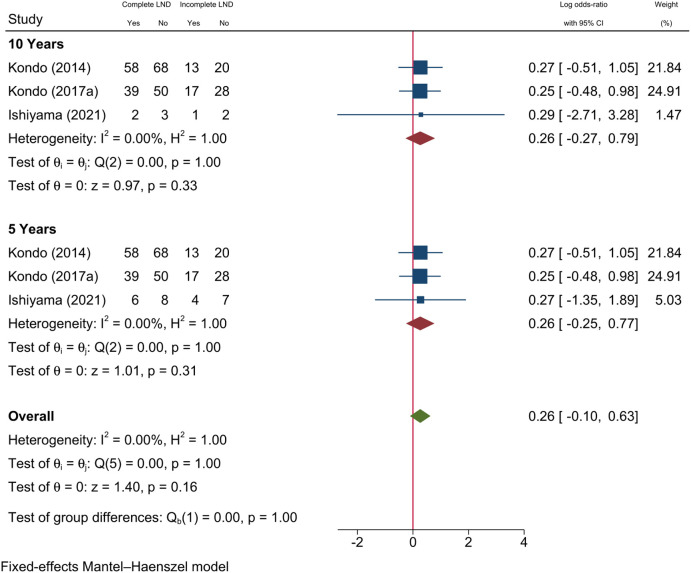
A forest plot showing the odds of recurrence-free survival between complete and incomplete LND stratified by follow-up.

Four studies were included in this meta-analysis which revealed no superior effect of complete LND when compared to incomplete LND in terms of 5-year [logOR = 0.37; 95% CI: −0.06-0.80; *I*^*2*^ = 0%] and 10-year [logOR = 0.29; 95% CI: −0.20-0.78; *I*^*2*^ = 0%] CSS (Supplementary Figure 8). Similarly, no significant change in the 5-year [logOR = 0.26; 95% CI: −0.25-0.77; *I*^*2*^ = 0%] and 10-year [logOR = 0.26; 95% CI: −0.27-0.79; *I*^*2*^ = 0%] RFS was noted between both groups (Supplementary Figure 9).

### Complications

**Figure 6.**
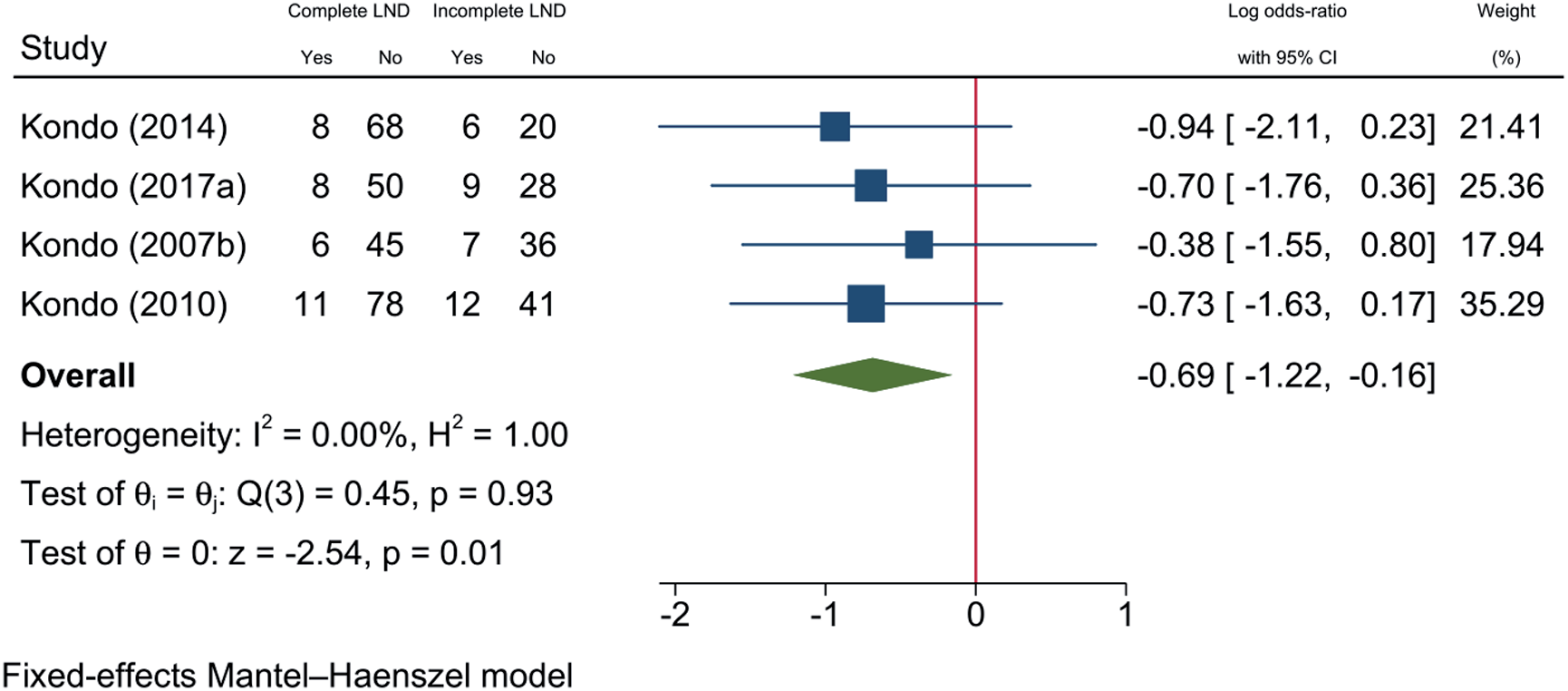
A forest plot showing the odds of cancer-specific mortality between complete and incomplete lymph node dissection. LND, lymph node dissection.

**Supplementary Figure 10.**
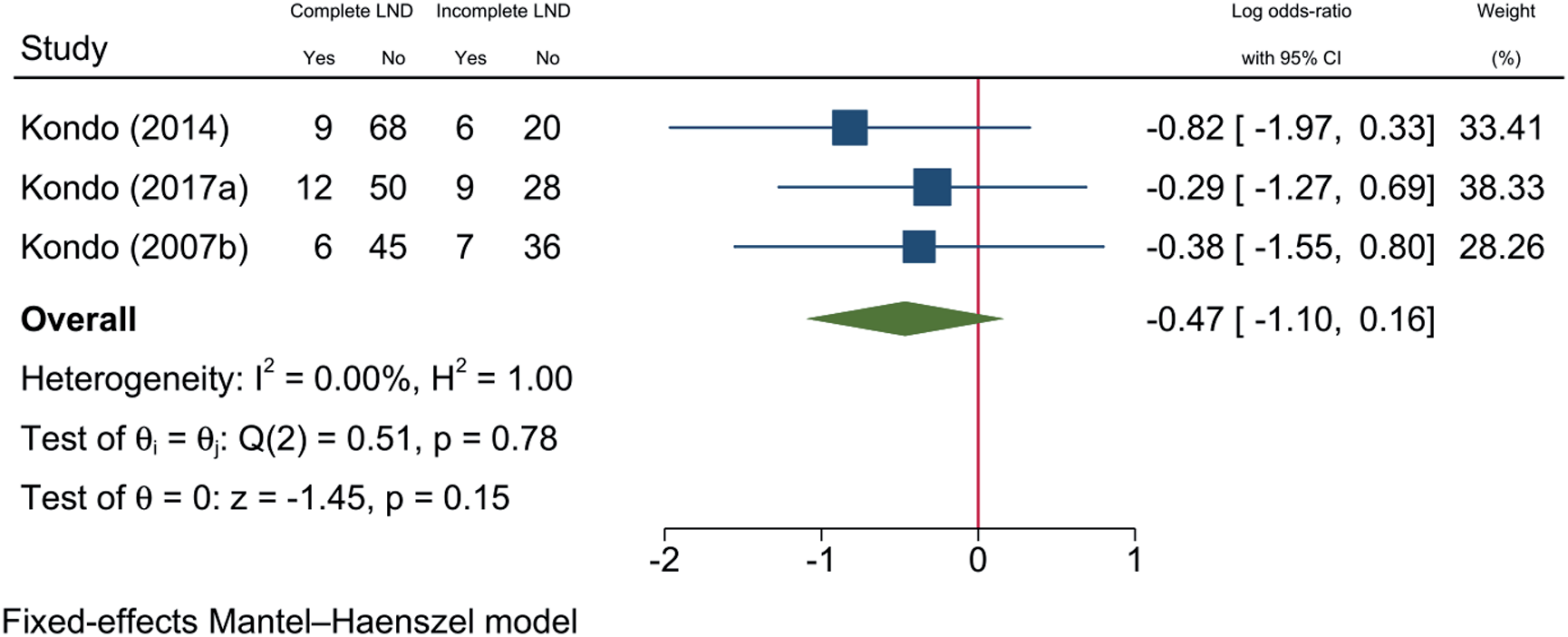
A forest plot showing the odds of recurrence between complete and incomplete LND.

Four and 3 studies assessed cancer-specific mortality and recurrence, respectively. The meta-analysis revealed a significantly lower risk of cancer-specific mortality in the complete LND arm as compared to the incomplete arm [logOR = −0.69; 95% CI: −1.22-−0.16; *I*^*2*^ = 0%] (Figure 6). However, no significant change in recurrence was noted between both groups [logOR = −0.47; 95% CI: −1.10-0.16; *I*^*2*^ = 0%] (Supplementary Figure 10).

### Negative Lymph Nodes vs. Positive Lymph Nodes

#### Prognosis:

**Figure 7.**
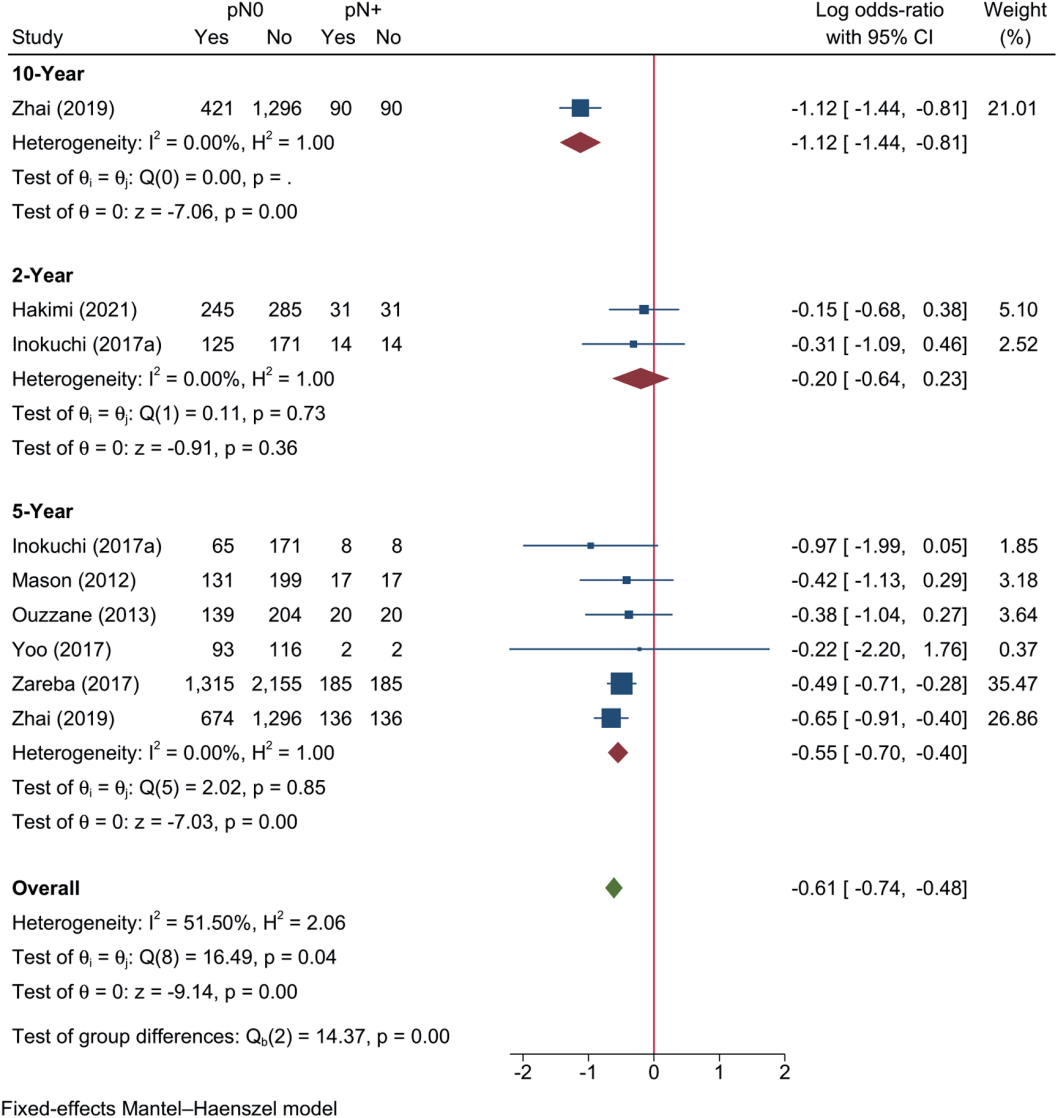
A forest plot showing the odds of overall survival between negative lymph node and positive lymph node groups stratified by follow-up. pN0, negative lymph node; pN+, positive lymph node.

**Figure 8.**
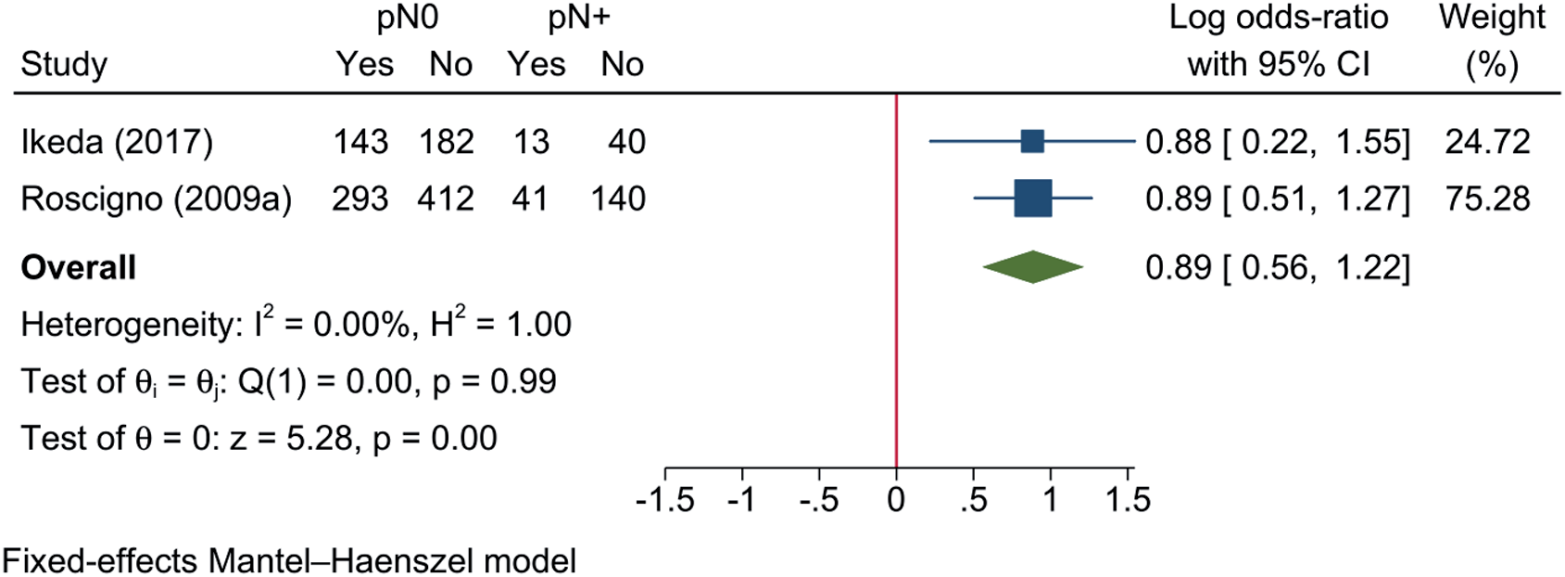
A forest plot showing the odds of disease-free survival between negative lymph node and positive lymph node groups stratified by follow-up. pN0, negative lymph node; pN+, positive lymph node.

**Figure 9.**
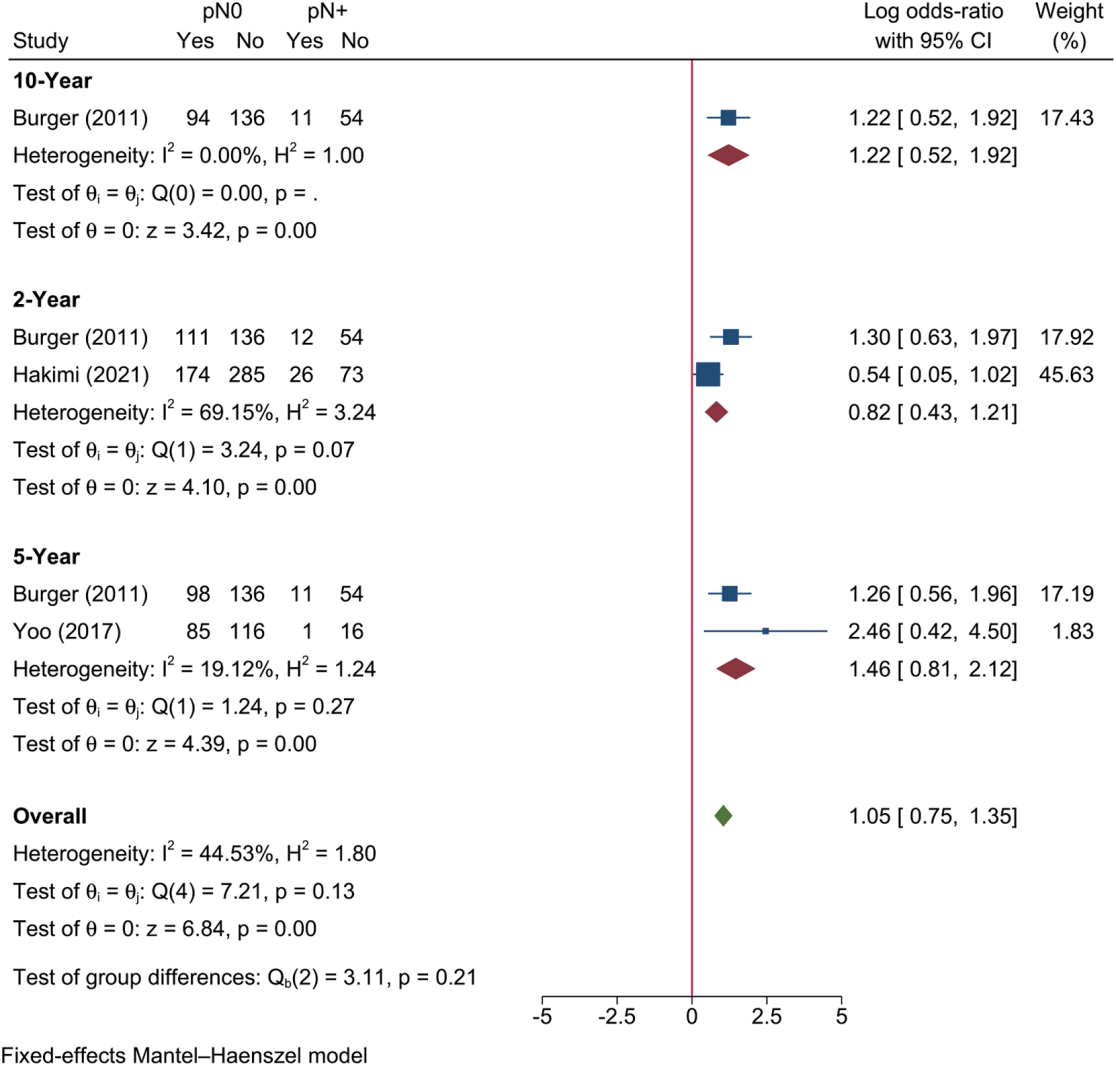
A forest plot showing the odds of recurrence-free survival between negative lymph node and positive lymph node groups stratified by follow-up. pN0, negative lymph node; pN+, positive lymph node.

**Figure 10.**
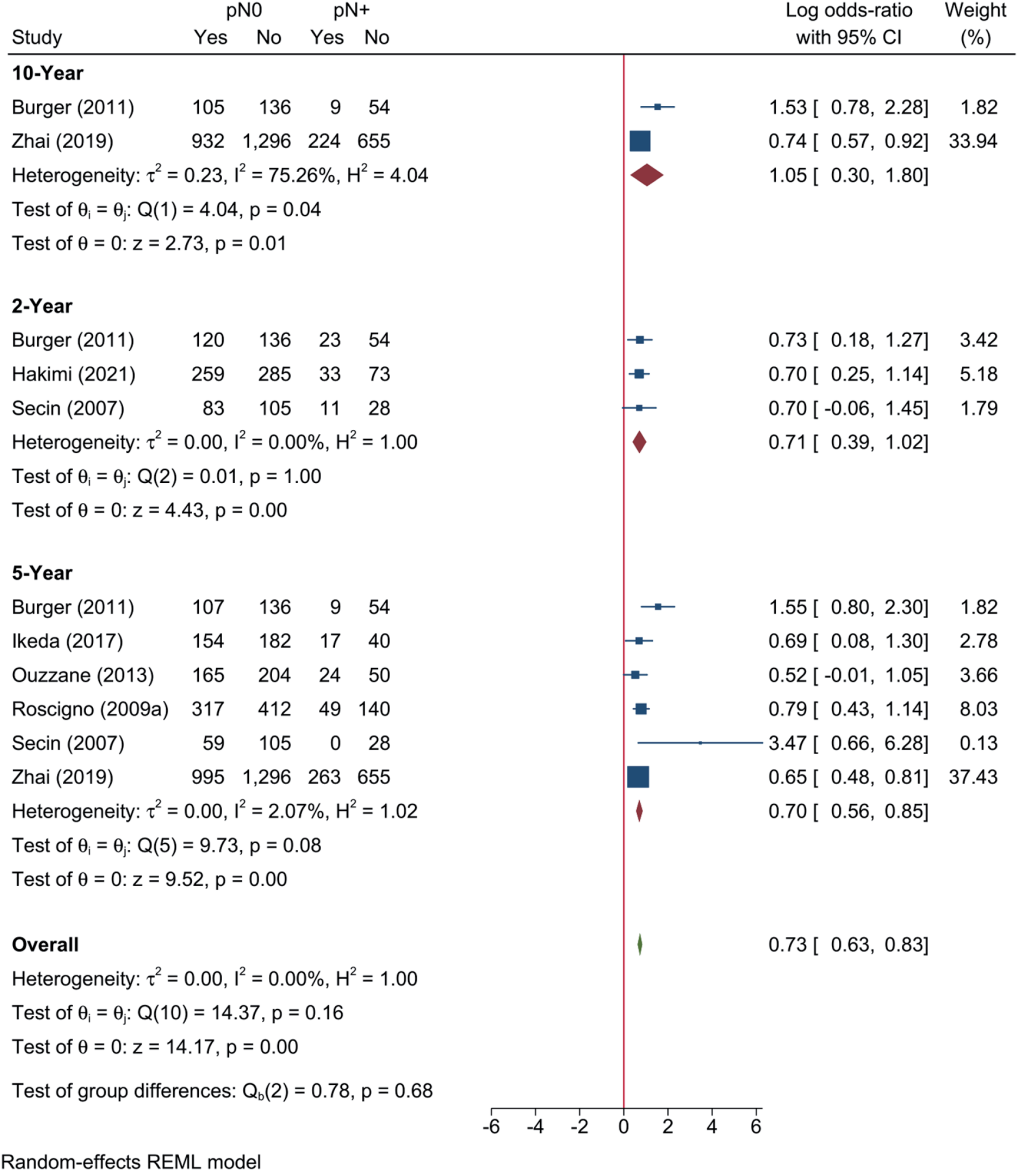
A forest plot showing the odds of cancer-specific survival between negative lymph node and positive lymph node groups stratified by follow-up. pN0, negative lymph node; pN+, positive lymph node. REML, restricted maximum likelihood method.

Seven studies compared the OS between patients who underwent LND with pN0 and pN+ nodal status. Overall, LND in pN0 patients revealed a significantly improved OS at 5 years as compared to pN+ patients [logOR = −0.55; 95% CI: −0.70-−0.40; *I*^*2*^ = 0%] (Figure 7). That being said, patients with positive node had significantly greater odds of DFS [logOR = 0.89; 95% CI: 0.56-1.22; *I*^*2*^ = 0%] (Figure 8). Consistently, patients with positive nodes had also greater odds of RFS at 2 years [logOR = 0.82; 95% CI: 0.43-1.21; *I*^*2*^ = 69.15%] and 5 years [logOR = 1.46; 95% CI: 0.81-2.12; *I*^*2*^ = 19.12%], respectively (Figure 9). Similarly, patients with positive nodes had significantly greater odds of 2-year [logOR = 0.71; 95% CI: 0.39-1.02; *I*^*2*^ = 0%], 5-year [logOR = 0.70; 95% CI: 0.56-0.85; *I*^*2*^ = 2.07%], and 10-year [logOR = 1.05; 95% CI: 0.30-1.80; *I*^*2*^ = 75.26%] CSS, respectively (Figure 10).

### Complications

No studies compared the complication rates between upper urothelial cancer patients with positive or negative nodes following LND.

## Discussion

The role of LND on the oncological outcomes of nephroureterectomy among UTUC patients has been studied extensively in the literature. Despite the availability of numerous studies, there is no clear consensus for numerous reasons. Firstly, there are no randomized controlled trials published to date on this topic. Secondly, most evidence is based on retrospective analyses that are bound to confounding bias. Thirdly, the majority of studies have small sample sizes, so statistically significant changes in survival outcomes are difficult to demonstrate. Finally, there is evident clinical heterogeneity in those studies with regards to the performed LND, specifically the nodal status (positive or negative), the number of resected nodes, or the extent of dissection (complete or incomplete).

This meta-analysis, by pooling survival data across 33 studies, provides the greatest evidence so far (by increasing the included sample size, and thus, increasing the power to detect significant changes) regarding the role of LND among UTUC patients who underwent nephroureterectomy. In our study, LND has shown a beneficial role in terms of 5-year OS, 5-year CSS, and 10-year CSS. However, the observed change in the odds of survival was minimal-to-moderate, favoring LND over no LND. Conversely LND did not result in any significant change in other survival outcomes, such as DFS and RFS. In terms of complications, patients who underwent LND had a significantly higher risk of high-grade complications (defined as complications of grade 3 or more based on the Clavien–Dindo classification) when compared to those who did not undergo LND. These observations are not consistent with the study of Chan et al^41^ who conducted a similar meta-analysis on UTUC patients who underwent LND during nephroureterectomy. The authors noted no prognostic role of LND on RFS, OS, or CSS as well as complications. However, they noted a potential benefit of LND on RFS in UTUC patients with muscle-invasive and advanced disease. This difference in our findings could be related to the difference in the included sample size since Chan et al^41^ included only 11 studies in their quantitative analysis compared to the 33 studies in our search. Additionally, in contrast to their study our analyses have no-to-minimal heterogeneity, compared to theirs (*I*^*2*^ > 50%). Of note, in 2017, the European Association of Urology published a systematic review on the potential benefit of LND during radical nephroureterectomy for UTUC.^42^ Similar to the study of Chan et al,^41^ they observed no significant role of LND on survival in terms of OS, CSS, RFS, and metastatic-free survival. Again, their analysis was based only on 9 studies, all of which had a high risk of bias, further limiting the applicability of their results when compared to ours. 

The impact of the extent of LND on the prognosis of UTUC has been minimally investigated and reported in the literature, and individual studies could not draw solid conclusions.^12-14,19^ Our meta-analysis reports similar findings of no prognostic role of complete LND when compared to incomplete dissection regarding CSS and RFS. However, our study highlights that complete LND can significantly lower the risk of cancer-specific mortality with no effect on disease recurrence. These findings are of high-to-moderate certainty (based on cohort studies) due to the lack of statistical heterogeneity, the inclusion of sufficient number of studies, and the standardized criteria for complete “dissection of all regional sites” and incomplete dissection where “not all sites were dissected.”

The nodal status of resected lymph nodes has shown to have great prognostic value. For instance, based on our analysis, pN0 patients have better 5-year OS. However, the statistical heterogeneity observed in this finding further limits its reliability. In contrast to this, pN+ patients have shown a significantly better prognosis in terms of DFS, RFS, and CSS (2, 5, and 10 year). This observation is novel and still warrants further confirmation by large-scale randomized controlled trials.

There are various methods to detect and manage sentinel LNs.^43^ These include molecular lymphatic mapping, which has shown promise in accurately identifying sentinel LNs with the disease through a focused histopathological evaluation of the suspected nodes and, therefore, has resulted in improvements in the diagnosis of even micrometastases.^44^ Another method is PET scanning which provides functional rather than structural visualization of suspected LNs. Unlike conventional computed tomography and magnetic resonance imaging, PET is able to successfully identify the occurrence of metastasis even in normal-sized LN with high diagnostic accuracy (sensitivity = 92%, specificity = 91%).^43^ Additionally, molecular LN analysis could alter the diagnostic process for sentinel LNs. Its efficacy lies in its ability to detect cancer-enhanced transcripts with very high sensitivity for detecting LN metastasis in solid tumors.^45^ That being said, it should be noted that these methods are implemented in bladder cancer, and their use in UTUC is yet to be established.

### Study Limitations and Future Directions

Our study provides the greatest body of evidence regarding the prognostic role of LND in UTUC with special regard to the extent of dissection and status of resected nodes. However, it has several limitations that further limit the reliability and generalizability of our findings. First, none of the included studies were randomized, making the certainty of our evidence low-to-very low. Second, in several analyses significant statistical heterogeneity was observed and this could correlate to the clinical heterogeneity of included populations as in the stage of UTUC. Thirdly, the quality of two-thirds of included studies is poor while the remaining ones are of fair quality. Fourthly, we did not perfect a meta-analysis based on the number of resected nodes. The meta-analysis of Choo et al^46^ highlighted that the increase in the number of nodes was predictive of improved CSS; however, an updated meta-analysis is needed in this regard since the previous review included only 6 studies. Finally, the site of UTUC could be of prognostic value in patients undergoing nephroureterectomy. For instance, a previous report has indicated that cancers at upper/middle ureter are associated with favored survival, while lower ureteral cancers have poor prognosis.^13^ This was not addressed in our review, and thus, future studies should address this point.

## Conclusion

In conclusion, lymph node dissection during nephroureterectomy among UTUC patients provides a protective role, when compared to no dissection, in terms of 5-year OS and 5-year and 10-year CSS. However, it is associated with higher risk of high-grade complications. Complete dissection is associated with lower risk of cancer-specific mortality. The status of dissected nodes plays an additional significant prognostic role, where node-positive patients have better survival outcome when compared to node-negative patients.

## Figures and Tables

**Table 1.** Baseline Characteristics of Included Studies

Author (YOP)	Country	Design	Sample Size					Follow-Up (Months)		Tumor Histology	Tumor Location	Surgery Type
			pN0	pNx	pN+	LND	Total	Mean	SD			
Abe (2008)^7^	Japan	Retrospective multicenter	139	146	27	166	312	49.58	33.53	UC [N = 282], others [N = 30]	Pelvis [N = 169], ureter [N = 120], pelvis and ureter [N = 23]	Open NU [N = 235], laparoscopic NU [N = 66], open nephrectomy [N = 11]
Abe (2010) ^21^	Japan	Retrospective multicenter	130	141	22	152	293	–	–	UC [N = 267], others [N = 26]	Pelvis [N = 157], ureter [N = 112], pelvis and ureter [N = 24]	Open NU [N = 220], laparoscopic NU [N = 66], open nephrectomy [N = 7]
Azawi (2017)^22^	Denmark	Retrospective multicenter	26	231	20	46	277	–	–	UC [N = 277]	–	–
Brausi (2007)^8^	Italy	Retrospective cohort	24	42	16	40	82	76.39	54.37	–	Pelvis [N = 47], ureter [N = 28], pelvis and ureter [N = 7]	–
Burger (2011)^9^	Germany	Retrospective multicenter	136	595	54	190	785	38.2	37.13	UC [N = 785]	–	Open RNU [N = 715], laparoscopic RNU [N = 70]
Dong (2019)^23^	Japan	Retrospective cohort	325	325	–	–	650	–	–	UC [N = 650]	Pelvis [N = 401], ureter [N = 249]	–
Furuse (2017)^24^	Japan	Retrospective cohort	–	16	–	61	77	64.2	44.6	UC [N = 77]	Pelvis [N = 40], ureter [N = 37]	–
Hakimi (2021)^10^	USA	Retrospective cohort	285	519	73	358	877	14.08	16.94	UC [N = 877]	–	–
Ikeda (2017)^11^	Japan	Retrospective cohort	182	177	40	222	399	50	53.57	UC [N = 372], others [N = 27]	Pelvis [N = 213], ureter [N = 186]	Open RNU [N = 296], laparoscopic RNU [N = 103]
Inokuchi (2017a)^25^	Japan	Retrospective cohort	171	626	26	197	823	49.26	31.86	UC [N = 808], others [N = 15]	Pelvis [N = 434], ureter [N = 375], pelvis and ureter [N = 14]	Open RNU [N = 505], laparoscopic RNU [N = 318]
Inokuchi (2017b)^26^	Japan	Retrospective multicenter	–	–	–	–	182	–	–	UC [N = 182]	Pelvis [N = 119], ureter [N = 63]	–
Kanno (2018)^28^	Japan	Retrospective cohort	–	32	–	32	64	–	–	UC [N = 64]	Pelvis [N = 45], ureter [N = 15], multiple [N = 4]	Laparoscopic RNU [N = 64]
Kanno (2022)^27^	Japan	Retrospective cohort	–	195	–	88	283	–	–	UC [N = 283]	Pelvis [N = 146], ureter [N = 120], pelvis and ureter [N = 17]	Laparoscopic RNU [N = 283]
Kikuchi (2014)^29^	Japan	Retrospective cohort	–	89	–	77	166	27.85	20.4	UC [N = 166]	Pelvis [N = 90], ureter [N = 76]	Open NU [N = 166]
Kondo (2014)^12^	Japan	Retrospective cohort	–	92	–	88; [complete LND = 68], [incomplete LND = 20]	180	46.33	47.28	UC [N = 180]	Pelvis [N = 180]	–
Kondo (2017)^13^	Japan	Retrospective cohort	–	76	–	78; [complete LND = 50], [incomplete LND = 28]	154	49.43	49.58	UC [N = 154]	Upper/middle ureter [N = 71], lower ureter [N = 83]	–
Kondo (2007)^30^	Japan	Retrospective cohort	–	88	–	81	169	48.82	44.1	UC [N = 169]	Pelvis [N = 100], ureter [N = 69]	ORNU [N = 146], RRNU [N = 7], RN [N = 5], segmental ureterectomy [N = 7], endoscopic ablation [N = 4]
Lee (2022)^15^	China	Retrospective cohort	–	463	–	195	658	–	–	–	Pelvis [N = 311], ureter [N = 223], pelvis and ureter [N = 121]	–
Lughezzani (2010)^31^	Canada	Retrospective cohort	1835	747	242	2077	2428	43.6	28.88	UC [N = 2824]	Pelvis [N = 1913], ureter [N = 911]	NU with cuff [N = 1951], NU without cuff [N = 873]
Mason (2012)^32^	Canada	Retrospective cohort	199	753	77	276	1029	–	–	UC [N = 1029]	Pelvis [N = 538], ureter [N = 250], pelvis and ureter [N = 213]	Laparoscopic NU [N = 446]
Miyake (1998)^34^	Japan	Retrospective cohort	–	37	–	35	72	49	–	UC [N = 72]	Pelvis [N = 40], ureter [N = 29], pelvis and ureter [N = 3]	–
Moschini (2017)^35^	Austria	Retrospective cohort	–	967	–	545	1512	48 [median]	–	UC [N = 1512]	–	Open RNU [N = 1007], laparoscopic RNU [N = 505]
Ouzzane (2013)^36^	France	Retrospective cohort	204	460	50	254	714	29.1	29.71	UC [N = 714]	Pelvis [N = 388], ureter [N = 236], multiple [N = 90]	–
Pearce (2016)^37^	USA	Retrospective cohort	–	14059	–	2560	16619	–	––	UC [N = 16619]	–	Open NU [N = 116 987], laparoscopic NU [N = 2638], robotic NU [N = 2286]
Roscigno (2009a)^17^	Italy	Retrospective cohort	412	578	140	552	1130	46.62	38.17	UC [N = 1130]	–	Open RNU [N = 924], laparoscopic RNU [N = 206]
Secin (2007)^38^	USA	Retrospective cohort	105	119	28	133	252	–	–	UC [N = 252]	–	–
Yoo (2017)^39^	Korea	Retrospective cohort	116	286	16	132	418	69	–	UC [N = 418]	Pelvis/proximal ureter [N = 206], mid-ureter [N = 49], distal ureter [N = 104], multiple [N = 59]	Open RNU [N = 184], minimal invasive RNU [N = 234]
Zareba (2017)^40^	USA	Randomized trial	2155	11546	771	2926	14 472	44.94 [among survivors]	32.92	UC [N = 14472]	Pelvis [N = 9936], ureter [N = 5436]	–
Zhai (2019)^16^	China	Retrospective cohort	1296	5317	665	1961	7278	–	–	UC [N = 7278]	Pelvis [N = 5032], ureter [N = 2246]	–
Kondo (2010)^14^	Japan	Retrospective cohort	–	98	21	119	119	46.04	46.27	UC [N = 119]	–	ONU [N = 102], retroperitoneoscopic NU + open distal ureter and bladder cuff removal [N = 8]
Ishiyama (2021)^19^	Japan	Retrospective multicenter	38	13	9	47	60	28.79	30.47	UC [N = 60]	Pelvis [N = 43], ureter [N = 17]	ORNU [N = 46]
Matsumoto (2020)^33^	Japan	Retrospective cohort	93	–	12	105	105	58.24	41.34	UC [N = 105], others [N = 1]	Pelvis/upper ureter [N = 69], lower ureter [N = 33], pelvis + ureter [N = 3]	ORNU [N = 105]
Roscigno (2009c)^18^	Italy	Retrospective multicenter	411	–	14	551	551	–	–	UC [N = 551]	–	ORNU [N = 463], LRNU [N = 88]

LND, lymph node dissection; pN+, positive lymph nodes; pN0, negative lymph nodes; pNx, no lymphadenectomy; UC, urothelial cancer; YOP, year of publication; RNU, Radical Nephroureterectomy; NU, Nephroureterectomy; ORNU, Open Radical Nephroureterectomy; RRNU, Right Radical Nephroureterectomy; RN, Radical Nephrectomy; ONU, Open Nephroureterectomy; LRNU, Left Radical Nephroureterectomy.

**Table 2.** The Characteristics of Included Upper Urothelial Cancer Patients Who Underwent Lymph Node Dissection

Author (YOP)	Age								Gender [Male]				
	pN0		pNx		pN+		LND						
	Mean	SD	Mean	SD	Mean	SD	Mean	SD	pN0	pNx	pN+	LND	Total
Abe (2008)^7^	–	–	–	–	–	–	–	–	–	–	–	–	312
Abe (2010)^21^	67.72	8.49	69.47	9.93	70.22	8.64	69.95	9.84	89	92	14	103	293
Azawi (2017)^22^	–	–	–	–	–	–	–	–	–	–	–	–	277
Brausi (2007)^8^	–	–	67.1^*^	–	–	–	67.8*	–	–	32	–	27	82
Burger (2011) ^9^	65.11	10.34	68.58	9.81	69.17	9.67	66.26	10.3	101	399	42	143	785
Dong (2019)^23^	–	–	–	–	–	–	–	–	196	183	–	–	650
Furuse (2017)^24^	–	–	73.67	7.35	–	–	69.79	7.67	–	10	–	44	77
Hakimi (2021)^10^	69.1	9.6	71.3	10.1	71.1	9.8	69.51	9.66	190	287	45	235	877
Ikeda (2017)^11^	66.35	8.22	69.3	10.46	68.35	10	66.71	8.58	141	135	31	185	399
Inokuchi (2017a)^25^	–	–	9.66	626	–	–	68.16	10.4	–	436	–	142	823
Inokuchi (2017b)^26^	–	–	–	–	–	–	–	–	–	–	–	–	182
Kanno (2018)^28^	–	–	70.6	8.4	–	–	70.2	8.4	–	21	–	23	64
Kanno (2022)^27^	–	–	74.65	8.22	–	–	71.44	9.04	–	134	–	67	283
Kikuchi (2014)^29^	–	–	74.29	11.5	–	–	67.57	8.41	–	53	–	59	166
Kondo (2014) ^12^	–	–	73.6	11.14	–	–	66.92	6.72	–	62	–	66	180
Kondo (2017)^13^	–	–	74.5	8.6	–	–	67.51	9.42	–	42	–	50	154
Kondo (2007)^30^	–	–	68.84	9.31	–	–	66.22	8.51	–	–	–	–	169
Lee (2022)^15^	–	–	69.8	10.8	–	–	67.7	10.6	–	196	–	94	658
Lughezzani (2010)^31^	70.8	–	72	–	72.3	–	70.97	–	1102	432	132	1234	2428
Mason (2012)^32^	69.9	10.8	67.3	10.6	66.7	9.2	69	10.46	139	460	55	194	1029
Miyake (1998)^34^	–	–	67	–	–	–	64	–	–	27	–	27	72
Moschini (2017)^35^	–	–	69.3	11.88	–	–	68.65	9.66	–	672	–	362	1512
Ouzzane (2013)^36^	68.95	11.2	68.65	12.64	69.1	9.92	68.97	10.94	140	316	28	168	714
Pearce (2016)^37^	–	–	–	–	–	–	–	–	–	8390	–	1631	16 619
Roscigno (2009a)^17^	68.66	10.63	69.27	10.27	69.15	12.04	68.78	11	–	–	–	–	1130
Secin (2007)^38^	68.94	9.77	68.95	11.26	66.57	15.62	68.45	11.23	64	86	16	80	252
Yoo (2017)^39^	63.5	10.2	64.1	10.3	61.9	7.4	63.3	9.9	27	80	6	33	418
Zareba (2017)^40^	68.65	12.61	71.65	11.12	69.65	12.63	68.91	12.62	1254	6599	411	1665	14472
Zhai (2019)^16^	–	–	72.4	10.8	–	–	70.7	10.6	–	3140	–	1155	7278
Kondo (2010)^14^	–	–	–	–	–	–	[Total = 67.58 [7]], [Complete LND = 66.57], [Incomplete LND = 69.49]	[Total = 9.85], [Complete LND = 9.34], [Incomplete LND = 10.62]	–	–	–	–	119
Ishiyama (2021)^19^	–	–	[No/Incomplete LND = 78.1]	[No/Incomplete LND = 3.55]	–	–	[Total = 78.5], [Complete LND = 78.88], [No/Incomplete LND = 78.1]	[Total = 3], [Complete LND = 2.33], [No/Incomplete LND = 3.55]	–	[No/Incomplete LND = 16]	–	[Total = 30], [Complete LND = 14], [No/Incomplete LND = 16]	60
Matsumoto (2020)^33^	–	–	–	–	–	–	71.65	9.77	–	–	–	75	105
Roscigno (2009c)^18^	67.86	10.63	–	–	67.15	12.04	67.75	10.97	–	–	–	–	551

LND, lymph node dissection; pN+, positive lymph nodes; pN0, negative lymph nodes; pNx, no lymphadenectomy; YOP, year of publication.

^*^Data presented as median.

**Table 3.** Quality Assessment of Nonrandomized Interventional Studies (Cohort and Case–Control) Using the Newcastle Ottawa Scale

Author (YOP)	Selection	Comparability	Exposure	Overall Quality
Azawi (2017)^22^	3	2	2	Fair
Dong (2019)^23^	3	2	2	Fair
Ikeda (2017)^11^	3	2	2	Fair
Kanno (2022)^27^	3	2	2	Fair
Kondo (2017)^13^	3	2	2	Fair
Lughezzani (2010)^31^	3	2	2	Fair
Miyake (1998)^34^	3	2	2	Fair
Ouzzane (2013)^36^	3	2	2	Fair
Secin (2007)^38^	3	2	2	Fair
Zhai (2019)^16^	3	2	2	Fair
Matsumoto (2020)^33^	3	2	2	Fair
Abe (2008)^7^	2	0	2	Poor
Abe (2010)^21^	2	0	2	Poor
Brausi (2007)^8^	3	0	2	Poor
Burger (2011)^9^	2	0	2	Poor
Furuse (2017)^24^	2	0	2	Poor
Hakimi (2021)^10^	2	0	2	Poor
Inokuchi (2017a)^25^	2	0	2	Poor
Inokuchi (2017b)^26^	2	0	2	Poor
Kanno (2018)^28^	2	0	2	Poor
Kikuchi (2014)^29^	3	0	2	Poor
Kondo (2014)^12^	2	0	2	Poor
Kondo (2007)^30^	2	0	2	Poor
Lee (2022)^15^	2	0	2	Poor
Mason (2012)^32^	3	0	2	Poor
Moschini (2017)^35^	2	0	2	Poor
Pearce (2016)^37^	2	0	2	Poor
Roscigno (2009a)^17^	2	0	2	Poor
Yoo (2017)^39^	3	0	2	Poor
Zareba (2017)^40^	2	0	2	Poor
Kondo (2010)^14^	2	0	2	Poor
Ishiyama (2021)^19^	2	0	2	Poor
Roscigno (2009b)^18^	3	0	2	Poor

YOP, year of publication.

**Supplementary Table 1.** The Detailed Search Strategy Modified Per Each Searched Database

Database	No	Search Query	Results
PubMed [Date of search: Sept 11, 2022]			
	#1	“lymph node*”	289160
	#2	excision OR removal OR dissection	1100020
	#3	#1 AND #2	70371
	#4	"Lymph Node Excision"[Mesh] OR Lymphadenectom*	61673
	#5	#3 OR #4	87926
	#6	“upper tract” OR “upper urinary tract”	10491
	#7	“urothelial cancer” OR “urothelial carcinoma*” OR “urothelial neoplasm” OR “transitional cell carcinoma”	31164
	#8	#6 AND #7	3789
	#9	Nephroureterectomy OR "Nephroureterectomy"[Mesh] OR "Ureteral Neoplasms"[Mesh]	7376
	#10	#8 OR #9	8820
	#11	#5 AND #10	467
Scopus [Date of search: Sept 11, 2022]			
	#1	ALL (“lymph node*”)	741527
	#2	ALL (excision) OR ALL (removal) OR ALL (dissection)	3345668
	#3	#1 AND #2	178804
	#4	ALL (Lymphadenectom*)	83002
	#5	#3 OR #4	210445
	#6	ALL (“upper tract”) OR ALL (“upper urinary tract”)	35246
	#7	ALL (“urothelial cancer”) OR ALL (“urothelial carcinoma*”) OR ALL (“urothelial neoplasm”) OR ALL (“transitional cell carcinoma”)	104082
	#8	#6 AND #7	13231
	#9	ALL (Nephroureterectomy)	8817
	#10	#8 OR #9	17378
	#11	#5 AND #10	892
Web of Science [Date of search: Sept 11, 2022]			
	#1	ALL=“lymph node*”	232878
	#2	ALL=excision OR ALL=removal OR ALL=dissection	1105211
	#3	#1 AND #2	46563
	#4	ALL=Lymphadenectom*	26167
	#5	#3 OR #4	62639
	#6	ALL=“upper tract” OR ALL=“upper urinary tract”	10673
	#7	ALL=“urothelial cancer” OR ALL=“urothelial carcinoma*” OR ALL=“urothelial neoplasm” OR ALL=“transitional cell carcinoma”	32852
	#8	#6 AND #7	4985
	#9	ALL=Nephroureterectomy	3887
	#10	#8 OR #9	6553
	#11	#5 AND #10	498
CENTRAL [Date of search: Sept 11, 2022]			
	#1	“lymph node*”	10886
	#2	excision OR removal OR dissection	38590
	#3	#1 AND #2	4790
	#4	Lymphadenectom*	1912
	#5	#3 OR #4	5564
	#6	“upper tract” OR “upper urinary tract”	575
	#7	“urothelial cancer” OR “urothelial carcinoma*” OR “urothelial neoplasm” OR “transitional cell carcinoma”	1622
	#8	#6 AND #7	193
	#9	Nephroureterectomy	128
	#10	#8 OR #9	219
	#11	#5 AND #10	22
Google Scholar [Date of search: Sept 11, 2022]			
	With all of the words	Lymph node Nephroureterectomy	
	With the exact phrase		
	With at least one of the words	Excision dissection removal	
	Total	Only the first 200 were retrieved and screened	200
